# Post-Quantum Security of COPA

**DOI:** 10.3390/e27090890

**Published:** 2025-08-23

**Authors:** Ping Zhang, Yutao Wang

**Affiliations:** 1 School of Computer Science, Nanjing University of Posts and Telecommunications, Nanjing 210023, China; 1023041136@njupt.edu.cn; 2 Advanced Cryptography and System Security Key Laboratory of Sichuan Province, Chengdu 610225, China

**Keywords:** COPA, post-quantum security, Simon’s algorithm, indistinguishable under quantum chosen-plaintext attack (IND-qCPA), existentially unforgeable under quantum chosen message attack (EUF-qCMA)

## Abstract

COPA is a notable authenticated online cipher and was one of the winning proposals for the CAESAR competition. Current works describe how to break the existentially unforgeable under quantum chosen message attack (EUF-qCMA) of COPA. However, these works do not demonstrate the confidentiality of COPA in the quantum setting. This paper fills this gap, considers the indistinguishable under quantum chosen-plaintext attack (IND-qCPA) security for privacy, and presents the first IND-qCPA security analysis of COPA. In addition, in order to effectively avoid the problems of quantum existential forgery attack and quantum distinguishing attack, we introduce an intermediate state doubling-point technology into COPA, restrict the associated data non-emptiness, and present an enhanced variant, called COPA-ISDP, to support the IND-qCPA and EUF-qCMA security. Our work is of great significance, as it provides a simple and effective post-quantum secure design idea to resist Simon’s attack.

## 1. Introduction

The rapid development of quantum technology in the Noisy Intermediate-Scale Quantum (NISQ) era poses significant threats to existing cryptographic schemes. Public-key cryptosystems based on integer factorization and discrete logarithm problems become vulnerable with Shor’s algorithm [1], accelerating the post-quantum cryptography standardization process. In contrast, symmetric-key cryptosystems are generally considered resistant to quantum attacks; consequently, research on their quantum security has remained limited until recently.

In recent years, as Simon’s algorithm [2], Bernstein-Vazirani algorithm [3], Deutsch-Jozsa algorithm [4], and other quantum algorithms [5,6,7,8] have realized the key recovery attacks and existential forgery attacks of structured symmetric-key ciphers, people have paid more attention to the quantum security of symmetric-key ciphers. Quantum algorithm is an important tool for carrying out quantum cryptanalysis. For example, Simon’s algorithm can find out the period of a function and carry out polynomial-time attack on the symmetric-key cipher or mode of operation with a special structure, so as to achieve exponential acceleration [2]. The Bernstein–Vazirani algorithm can determine the linear structure of symmetric-key cryptography and attack symmetric-key cryptography with a linear structure in polynomial time, so as to achieve exponential acceleration [3].

Existing studies on the post-quantum security of symmetric-key cryptography mainly focus on block ciphers and modes of operation on block ciphers. Post-quantum security analysis of block ciphers mainly focuses on Feistel [9,10,11], generalized Feistel [12], (tweakable) Even-Mansour cipher [13,14], Key-Alternating Ciphers (KACs) [15], Tweakable Block Ciphers (TBCs) [16], LRW [17], and other structures. Post-quantum security analysis of modes of operation on block ciphers mainly focuses on encryption modes, authentication modes or message authentication codes, and authenticated encryption (AE) modes [6,16,17,18,19,20,21,22].

The quantum security analysis is based on quantum superposition model, where the adversary is allowed to access quantum oracles with quantum superposition states (i.e., the quantum adversary has access to the quantum encryption oracle with quantum superposition states of the plaintext and returns the superposition states of the ciphertext). In 2016, Anand et al. considered some encryption modes of operation (including CBC, CFB, OFB, CTR, and XTS), presented a quantum security definition called IND-qCPA for privacy (which is unlike the classical IND-CPA security and allows for quantum encryption queries), and showed that most of these modes exhibit classical IND-CPA security but IND-qCPA insecurity [23]. In 2017, Kaplan et al. considered authentication modes and AE modes, focused on the authenticity (existential unforgeability under quantum chosen-message attacks, EUF-qCMA) security, and implemented quantum forgery attacks on authentication modes (such as CBC-MAC, PMAC and GMAC) and AE modes (such as GCM, OCB, CLOC, AEZ, COPA, OTR, POET, OMD, and Minalpher) by Simon’s algorithm [17]. In 2021, Guo et al. used Grover’s algorithm and the Grover-meets-Simon algorithm to realize quantum forgery attacks on many beyond-birthday-bound secure MACs (such as SUM-ECBC-like MAC, and PMAC-Plus-like MAC) [6]. In 2022, Maram et al. considered the quantum security of OCB, and presented the IND-qCPA security and universal unforgeability of OCB variants [19]. In 2023, Nan et al. used Simon’s algorithm and the Grover-meets-Simon algorithm to implement quantum forgery attacks on pEDM, PDM* and nEHtMp [24]. Lang and Lucks focused on the post-quantum security of classical authenticated encryption schemes, including the generic SIV mode, GCM mode, and EAX mode [22]. In 2025, Zhang et al. proposed a novel quantum query model tailored for AE with associated data (AEAD) and analyzed the relationships between different quantum IND-CPA security notions [20].

COPA, designed by Andreeva et al., is a classical authenticated encryption (AE) scheme supporting associated data and a winner of the prestigious CAESAR competition [25]. It offers parallelizable processing with birthday-bound security when instantiated with a strong pseudorandom permutation. However, its security guarantees were fundamentally challenged by Xu et al. (2021) [26], who demonstrated quantum existential forgery attacks on both COPA and AES-COPA. By leveraging Simon’s algorithm to identify periods in the tag generation function through superposition queries, they successfully forged valid tags for new messages. Critically, while this work exposed vulnerabilities in authenticity under quantum attacks, it left a significant gap: the confidentiality of COPA in quantum settings remained unexplored. This omission is particularly consequential given that

- Post-quantum security demands joint guarantees for both privacy (IND-qCPA) and integrity (EUF-qCMA);
- NIST’s migration guidelines explicitly require quantum-resistant confidentiality;
- No prior work has established whether COPA’s encryption mechanism resists quantum plaintext recovery attacks.

**Our contribution.** Our work directly addresses this critical knowledge gap by providing the first formal analysis of COPA’s privacy in quantum settings. Our contribution includes

1. The first IND-qCPA security analysis of COPA in quantum settings, establishing its confidentiality guarantees under quantum chosen-plaintext attacks.
2. COPA-ISDP: An enhanced variant achieving joint IND-qCPA and EUF-qCMA security via a mandatory non-empty associated data and a novel intermediate state doubling-point technique.
3. Efficient security hardening that
  - Preserves COPA’s parallelizability and efficiency.
  - Breaks input/tag invariance using finite field double-multiplication.
  - Mitigates quantum attacks (e.g., Simon’s algorithm).

Compared with Xu et al.’s prior work [26], our work fundamentally differs in both scope and methodology, and provides significant advancements beyond their results:

1. Distinct Security Goals: Confidentiality vs. Authenticity—Xu et al. (2021) [26] focused exclusively on EUF-qCMA security (authenticity), demonstrating how Simon’s algorithm can forge authentication tags via superposition queries. Our work addresses the critical gap they left: IND-qCPA security (confidentiality). We provide the first formal proof that COPA’s encryption mechanism fails to achieve IND-qCPA under quantum chosen-plaintext attacks. This establishes that COPA leaks plaintext information in quantum settings—a vulnerability that has not been addressed by prior work.
2. Formalized Security Framework vs. Concrete Attacks—Xu et al. employed an attack-driven approach: They designed a specific quantum forgery attack but did not generalize COPA’s security properties. Our work develops a rigorous reductionist framework: We prove IND-qCPA insecurity via a game-based reduction to the quantum pseudorandom permutation (qPRP) security of the underlying block cipher (Section 3).
3. Constructive Remedy with Provable Guarantees—Xu et al. exposed a vulnerability (authenticity), but they proposed no solution. In contrast, we introduce COPA-ISDP, an enhanced variant that achieves both IND-qCPA and EUF-qCMA security via intermediate state doubling-point and mandatory non-empty associated data. In addition, we provide joint security proofs for IND-qCPA and EUF-qCMA in the quantum random oracle model.
4. Broader Impact: A Design Paradigm—Our work transcends COPA-specific analysis. The ISDP technique offers a generic countermeasure against quantum invariance attacks, and this is validated in our repair of COPA and can be extended to symmetric primitives.

The comparison between Xu et al.’s work [26] and our work is shown in Table 1.

**Organization of this paper. **Section 2 presents some preliminaries. Section 3 shows quantum attacks on COPA. Section 4 introduces an enhanced variant of COPA. Section 5 concludes this paper.

## 2. Preliminaries

This section introduces some basic syntax, including permutation, block cipher, and authenticated encryption with associated data (AEAD); some basic quantum circuit models, including the permutation circuit, block cipher circuit, and AEAD circuit; and some quantum security models, including the IND-qCPA security model for privacy and the EUF-qCMA security model for authenticity.

### 2.1. Basic Syntax

Let $P:{\left\{0,1\right\}}^{n}\to {\left\{0,1\right\}}^{n}$ be an *n*-bit permutation and $P^{-1}$ be the inverse of *P*. Let $R:{\left\{0,1\right\}}^{n}\to {\left\{0,1\right\}}^{n}$ be an *n*-bit function.

A block cipher with a key space $K={\left\{0,1\right\}}^{k}$ and a message space ${\left\{0,1\right\}}^{n}$ is a map $E:{\left\{0,1\right\}}^{k}\times {\left\{0,1\right\}}^{n}\to {\left\{0,1\right\}}^{n}$ such that for any key K∈K, $E_{K}\left(\cdot \right)$ is an *n*-bit permutation and $D_{K}=E_{K}^{-1}$ is the inverse of $E_{K}$.

An AEAD scheme consists of the encryption algorithm E and decryption algorithm D. The syntax is shown as follows:

$$\begin{array}{cc} \; & \left(C,T\right)\leftarrow E\left(K,N,A,M\right)=E_{K}\left(N,A,M\right)\\ \; & M/⊥\leftarrow D\left(K,N,A,C,T\right)=D_{K}\left(N,A,C,T\right), \end{array}$$

where K,N,A,M,C,T are the key, nonce, associated data, plaintext, ciphertext, and authentication tag, respectively. Moreover, for any N,A, and *M*, $D_{K}\left(N,A,E_{K}\left(N,A,M\right)\right)=M$ holds.

### 2.2. Quantum Circuit Models

Unitary Circuit Model: Let *f* be any function. In the superposition query, the adversary chooses |x〉|y〉 and receives |x〉|y⊕f(x)〉, i.e.,

$$\begin{array}{c} U_{f}:|x\rangle |y\rangle \to |x\rangle |y⊕f\left(x\right)\rangle . \end{array}$$

If *f* is a permutation *P* or a block cipher $E_{K}$, the circuit is also called as quantum permutation circuit or block cipher circuit.

AEAD Circuit Model: Let Π=(E,D) be an AEAD which consists of encryption and decryption unitary circuits. In the superposition query to E, the adversary chooses |K〉|N〉|A〉|M〉|y〉 and receives $|K\rangle |N\rangle |A\rangle |M\rangle |y⊕E_{K}\left(N,A,M\right)\rangle$, i.e.,

$$\begin{array}{c} U_{E}:|K\rangle |N\rangle |A\rangle |M\rangle |y\rangle \to |K\rangle |N\rangle |A\rangle |M\rangle |y⊕E_{K}\left(N,A,M\right)\rangle . \end{array}$$

In the superposition query to D, the adversary chooses |K〉|N〉|A〉|C〉|T〉|y〉 and receives $|K\rangle |N\rangle |A\rangle |C\rangle |T\rangle |y⊕D_{K}\left(N,A,C,T\right)\rangle$, i.e.,

$$\begin{array}{c} U_{D}:|K\rangle |N\rangle |A\rangle |C\rangle |T\rangle |y\rangle \to |K\rangle |N\rangle |A\rangle |C\rangle |T\rangle |y⊕D_{K}\left(N,A,C,T\right)\rangle . \end{array}$$

### 2.3. Simon’s Algorithm

Simon’s algorithm (Simon, 1997 [2]) is an efficient quantum algorithm for settling the following Simon’s problem, with a complexity of O(n):

**Simon’s Problem**: Given a function $f:{\left\{0,1\right\}}^{n}\to {\left\{0,1\right\}}^{n}$ and a promise that there exists a non-zero $s\in {\left\{0,1\right\}}^{n}$ such that, for any two distinct $x,y\in {\left\{0,1\right\}}^{n}$, f(x)=f(y)⟺x=y⊕s, the goal is to find *s*.

### 2.4. Quantum Security Models

For block cipher, we consider quantum-secure pseudorandom permutation (qPRP) and quantum-secure pseudorandom function (qPRF) models [19].

**Definition** **1** (qPRP [19])**.** *A block cipher $E_{K}:{\left\{0,1\right\}}^{n}\to {\left\{0,1\right\}}^{n}$ is a quantum-secure pseudorandom permutation (qPRP) if no efficient quantum adversary A making quantum queries can distinguish between a truly random permutation $P:{\left\{0,1\right\}}^{n}\to {\left\{0,1\right\}}^{n}$ and $E_{K}$ for a uniformly random secret key $K\overset{\$}{\leftarrow }{\left\{0,1\right\}}^{k}$.*

*The advantage of A in distinguishing $E_{K}$ from the random permutation π is defined as*

$$\begin{array}{c} Adv_{E}^{qPRP}\left(A\right)=\left|Pr\left[A^{E_{K}}=1\right]-Pr\left[A^{P}=1\right]\right|, \end{array}$$

*where $A^{O}$ denotes that A has quantum oracle access to $O\in \{E_{K},P\}$. Then, we have $E_{K}$ as a qPRP if $Adv_{E}^{qPRP}\left(A\right)$ is negligible for any polynomial-time quantum adversary A.*

**Definition** **2** (qPRF [19])**.** *A function $E_{K}:{\left\{0,1\right\}}^{n}\to {\left\{0,1\right\}}^{n}$ is a quantum-secure pseudorandom function (qPRF) if no efficient quantum adversary A making quantum queries can distinguish between a truly random function $R:{\left\{0,1\right\}}^{n}\to {\left\{0,1\right\}}^{n}$ and the function $E_{K}$ for a uniformly random secret key $K\overset{\$}{\leftarrow }{\left\{0,1\right\}}^{k}$.*

*The advantage of A in distinguishing $E_{K}$ from the random function R is defined as*

$$\begin{array}{c} Adv_{E}^{qPRF}\left(A\right)=\left|Pr\left[A^{E_{K}}=1\right]-Pr\left[A^{R}=1\right]\right|, \end{array}$$

*where $A^{O}$ denotes that A has quantum oracle access to $O\in \{E_{K},R\}$. Then, we have $E_{K}$ as a quantum-secure PRF if $Adv_{E}^{qPRF}\left(A\right)$ is negligible for any polynomial-time quantum adversary A.*

**Lemma** **1** (qPRP-qPRF Switching Lemma [27])**.** *Let R and P denote quantum oracles of a random function from ${\left\{0,1\right\}}^{n}$ to ${\left\{0,1\right\}}^{n}$ and an n-bit random permutation, respectively. Let A be an oracle-aided quantum algorithm that makes, at most, q quantum queries. Then, it holds that*

$$\begin{array}{c} Adv_{E}^{qPRP}\left(A\right)\leq Adv_{E}^{qPRF}\left(A\right)+O\left(q^{3}/2^{n}\right). \end{array}$$

For AEAD, we consider indistinguishable under quantum chosen-plaintext attack (IND-qCPA) for privacy and existentially unforgeable under chosen message attack (EUF-qCMA) for authenticity [19].

**Definition** **3** (IND-qCPA [19])**.** *An AEAD scheme Π=(E,D) is indistinguishable under quantum chosen-plaintext attack (IND-qCPA secure) if there is not an efficient quantum adversary that is able to win the following game, except with $\frac{1}{2}+\epsilon$ probability, where ϵ is a negligible value.*

1. *Key generation stage. $K\overset{\$}{\leftarrow }K$ and $b\overset{\$}{\leftarrow }\left\{0,1\right\}$ are randomly drawn by the challenge.*
2. *Query stage. The quantum adversary A is allowed to make two types of queries in any order.*
  - *Encryption queries. The challenge first randomly chooses a nonce N and forwards it to A. The adversary A chooses a plaintext-AD pair (M,A) in superposition, and the challenger encrypts (N,A,M) with the classical nonce N and returns the output (C,T) to A.*
  - Challenge query. The challenger picks a random nonce N once more and forwards it to A. Afterwards, A chooses two same size classical plaintext-AD pairs $\left(M_{0},A_{0}\right)\neq \left(M_{1},A_{1}\right)$ and forwards them to the challenger, which in turn encrypts $\left(N,A_{b},M_{b}\right)$ with the previous chosen classical nonce N. The output $\left(C^{*},T^{*}\right)$ is returned to A.
3. Guess stage. The adversary decides whether $\left(C^{*},T^{*}\right)$ is encrypted from $(N,A_{0},$ $M_{0})$ or $\left(N,A_{1},M_{1}\right)$, outputs a bit $b^{\prime }$, and wins if $b=b^{\prime }$.

*The IND-qCPA advantage of an adversary A against an AEAD scheme *Π *is defined as*

$$\begin{array}{c} Adv_{\Pi }^{IND-qCPA}\left(A\right)=\left|Pr\left[A\;wins\right]-\frac{1}{2}\right|. \end{array}$$

**Definition** **4** (EUF-qCMA [19])**.** *An AEAD scheme Π=(E,D) is existentially unforgeable under chosen message attack (EUF-qCMA secure) if there is no efficient quantum adversary that is able to win the following game, except with with a negligible probability.*

1. *Key generation stage. $K\overset{\$}{\leftarrow }K$ is randomly drawn by the challenge.*
2. *Query stage. The quantum adversary A is allowed to make encryption queries.*
  - *Encryption queries. The challenge first randomly chooses a nonce N and forwards it to A. The adversary A chooses a plaintext–AD pair (M,A) in superposition, and the challenger encrypts (N,A,M) with the classical nonce N and returns the output (C,T) to A.*
3. *Forgery stage. After making q encryption queries, A produces q+1 classical tuples (N,A,C,T) with any nonce N of its choice, and wins if, for each tuple, we have $D_{K}\left(N,A,C,T\right)\neq ⊥$.*

*The EUF-qCMA advantage of an adversary A against an AEAD scheme *Π *is defined as*

$$\begin{array}{c} Adv_{\Pi }^{EUF-qCMA}\left(A\right)=Pr\left[A\;wins\right]. \end{array}$$

## 3. Quantum Attacks on COPA

COPA, designed by Andreeva et al., is a classical birthday-bound secure block-cipher-based AEAD mode offering nonce-misuse resistance [25]. However, classical forgery attacks against COPA have been identified by scholars, though with limited success probability. Subsequently, Xu et al. demonstrated quantum forgery attacks on COPA using Simon’s algorithm, reducing the query complexity to O(n) and achieving near-100% success probability [26].

While existing works compromise COPA’s unforgeability in the quantum setting, they do not address its confidentiality under quantum attacks. This section provides a comprehensive post-quantum security analysis of COPA, covering both confidentiality and authenticity.

### 3.1. Specifications of COPA

Let *K*, *A*, *M*, *C*, and *T* denote the key, associated data, plaintext, ciphertext, and authentication tag, respectively. Define $E_{K}$ as a block cipher under key *K*. The associated data *A* is partitioned into *a* *n*-bit blocks A[1],…,A[a] or $A\left[a\right]||10^{*}$ and the plaintext *M* is divided into *d* *n*-bit blocks M[1],…,M[d]. Compute $L=E_{K}\left(0^{n}\right)$, and let $V={\mathrm{PMAC}1}_{K}\left(A\right)$ (where V=0 if *A* is empty). In addition to this, the generation of the mask sequence ${\left\{2^{i}\cdot 3^{j}\cdot L\right\}}_{i,j\in N}$ is implemented using the doubling-point technique. The so-called doubling-point technique involves using the double-multiplication operation of elements on a finite field to calculate new elements so that the operation speed is fast and highly efficient. For example, for $3\cdot a=2\cdot a⊕a,2\cdot 3\cdot a=2\cdot \left(2\cdot a⊕a\right),2^{2}\cdot a=2\cdot 2a,\cdots$.

**Figure 1:**
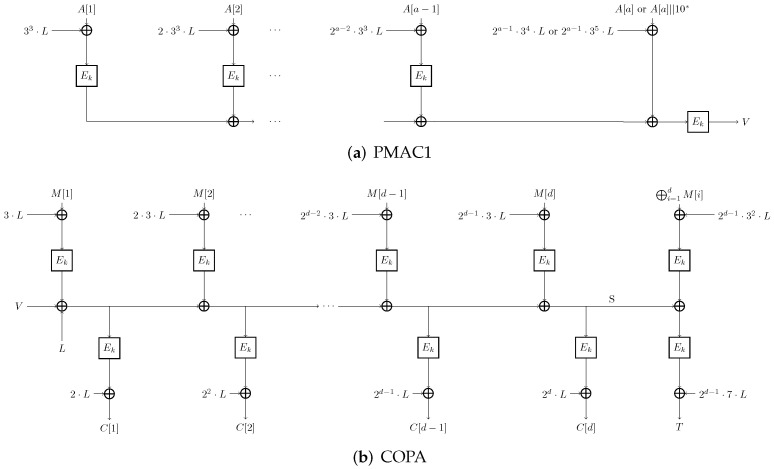
The overview of COPA.

The COPA encryption algorithm takes (K,A,M) as input and outputs (C,T), while the decryption algorithm takes (K,A,C,T) as input and outputs *M* or ⊥ (indicating failure). Figure 1 illustrates the COPA structure, with detailed procedures for PMAC1, encryption, and decryption provided in Algorithms 1, 2 and 3, respectively.

    **Algorithm 1** PMAC1 Algorithm: $PMAC1_{K}\left(A\right)$
**Input:** Key *K* and associated data *A*
**Output:** MAC tag *V*
if *A* is empty
   **return** V=0
else
   $L\leftarrow E_{K}\left(0^{n}\right)$
   Partition *A* into A[1]∥⋯∥A[a], where |A[i]|=n for 1≤i≤a−1,0<|A[a]|≤n
   for i=1 to a−1
       $X\left[i\right]\leftarrow A\left[i\right]⊕2^{i-1}\cdot 3^{3}\cdot L$
       $S\left[i\right]\leftarrow E_{K}\left(X\left[i\right]\right)$
   if |A[a]|=n
       $\Sigma \leftarrow S_{1}⊕S_{2}⊕\cdots ⊕S_{a-1}⊕A\left[a\right]$
       $V=E_{K}\left(\Sigma ⊕2^{a-1}\cdot 3^{4}\cdot L\right)$
   else
       $\Sigma \leftarrow S_{1}⊕S_{2}⊕\cdots ⊕S_{a-1}⊕A\left[a\right]10^{*}$
       $V=E_{K}\left(\Sigma ⊕2^{a-1}\cdot 3^{5}\cdot L\right)$
   **return** *V*


    **Algorithm 2** The encryption algorithm: $COPA.E_{K}\left(A,M\right)$
**Input:** Key *K*, associated data *A*, and plaintext *M*
**Output:** Ciphertext *C* and tag *T*
Partition *M* into M[1]∥⋯∥M[d], where |M[i]|=n,1≤i≤d


$V=PMAC1_{K}\left(A\right)$




$L=E_{K}\left(0^{n}\right)$




U[0]=V⊕L


for i=1 to *d*
    $S\left[i\right]\leftarrow E_{K}\left(M\left[i\right]⊕2^{i-1}\cdot 3\cdot L\right)$
    U[i]←U[0]⊕S[1]⊕S[2]⊕⋯⊕S[i]
    $C\left[i\right]\leftarrow E_{K}\left(U\left[i\right]\right)⊕2^{i}\cdot L$


C←C[1]C[2]⋯C[d]




S=U[d]




$\Sigma ={⨁}_{1\leq i\leq d}M\left[i\right]$




$T=E_{K}\left(E_{K}\left(\Sigma ⊕2^{d-1}\cdot 3^{2}\cdot L\right)⊕S\right)⊕2^{d-1}\cdot 7\cdot L$


**return** C||T


    **Algorithm 3** The decryption algorithm: $COPA.D_{K}\left(A,C||T\right)$
**Input:** Key *K*, associated data *A*, ciphertext *C*, and tag *T*
**Output:** Plaintext *M* or ⊥
Partition *C* into C[1]∥⋯∥C[d], where |C[i]|=n,1≤i≤d


$V=PMAC1_{K}\left(A\right)$




$L=E_{K}\left(0^{n}\right)$




U[0]=V⊕L


for i=1 to *d*
    $U\left[i\right]\leftarrow E_{K}^{-1}\left(C\left[i\right]⊕2^{i}\cdot L\right)$
    $M\left[i\right]\leftarrow E_{K}^{-1}\left(U\left[i\right]⊕U\left[i-1\right]\right)⊕2^{i-1}\cdot 3\cdot L$


M←M[1]M[2]⋯M[d]




S=U[d]




$\Sigma ={⨁}_{1\leq i\leq d}M\left[i\right]$




$T^{\prime }=E_{K}\left(E_{K}\left(\Sigma ⊕2^{d-1}\cdot 3^{2}\cdot L\right)⊕S\right)⊕2^{d-1}\cdot 7\cdot L$


if $T^{\prime }=T$
    **return** *M*
else
    **return** ⊥


### 3.2. IND-qCPA Security Analysis of COPA

If quantum superposition queries are allowed, a polynomial time attack is proposed against the confidentiality of COPA.

Considering a function $f_{A}:{\left\{0,1\right\}}^{n}\to {\left\{0,1\right\}}^{n}$, for input (K,A,M||M) and its output (C[1]||C[2],T), one has

$$\begin{array}{c} C\left[2\right]=f_{A}\left(M\right)=E_{K}\left(E_{K}\left(M⊕3\cdot L\right)⊕V⊕L⊕E_{K}\left(M⊕2\cdot 3\cdot L\right)\right)⊕2^{2}\cdot L. \end{array}$$

We found that $f_{A}$ is a periodic function with period s=3·L⊕2·3·L=5·L. Using Simon’s algorithm, we can recover the state *L* in a polynomial time O(n).

Through observation, we found that if M[1]=3·L, according to $L=E_{K}\left(0^{n}\right)$ and let $V=0^{n}$, we have C[1]=L⊕2·L=3·L.

Therefore, to achieve IND-qCPA attack, we perform the following steps:

In the challenge query stage, the challenge chooses a single-block message $M_{0}=3\cdot L$ and a random message $M_{1}\neq M_{0}$ (no associated data).

Upon receiving the response $\left(C^{*},T^{*}\right)$ from the challenger, the adversary A outputs bit $b^{\prime }=0$ if $C^{*}=3\cdot L$, and outputs $b^{\prime }=1$ otherwise.

In the guess stage, the adversary A easily decides whether $\left(C^{*},T^{*}\right)$ is encrypted from $M_{0}$ or $M_{1}$, i.e., the probability that A wins is 1. This is because of the following factors:

If $M_{0}=3\cdot L$ was encrypted by the challenger, then $C^{*}=E_{K}\left(E_{K}\left(0^{n}\right)⊕L\right)⊕2\cdot L=3\cdot L$.

If $M_{1}\neq M_{0}$ was encrypted by the challenger, then $C^{*}=E_{K}\left(E_{K}\left(M_{1}⊕3\cdot L\right)⊕L\right)⊕2\cdot L$. According to PRP/qPRP security, $Pr[C^{*}=3\cdot L]$ is negligible.

It follows that the IND-qCPA advantage of an adversary A against COPA is

$$\begin{array}{c} Adv_{COPA}^{IND-qCPA}\left(A\right)=\left|Pr\left[A\;wins\right]-\frac{1}{2}\right|=\frac{1}{2}. \end{array}$$

Therefore, our attack succeeds with high probability.

### 3.3. EUF-qCMA Security Analysis of COPA

Xu et al. found the function period of the authentication tag of COPA using Simon’s algorithm and presented an unforgeability attack that breaks the EUF-qCMA security of COPA [26]. We briefly describe the attack process as follows:

In the query stage, the quantum adversary A makes one encryption query (A,M||M) and utilizes it to construct a periodic function $f_{A}\left(M\right)$. Then, using Simon’s algorithm, we obtain $L=E_{K}\left(0^{n}\right)=s/5$, where *s* is the period of $f_{A}\left(M\right)$. (In fact, Xu et al. utilized one encryption query to construct a periodic function of the authentication tag, and then recovered $L=E_{K}\left(0^{n}\right)$. Please refer to their paper for details). Furthermore, A chooses associated data $A=A\left[1\right]||A\left[2\right]||\cdots ||A\left[a\right](A\left[a\right]||10^{*})$ and a message M=M[1]||M[2]||⋯||M[d] in the superposition, and the challenger encrypts (M,A) and returns the output (C=C[1]||C[2]||⋯||C[d],T) to A, where $C\left[i\right]=E_{K}\left({⨁}_{1\leq j\leq i}E_{K}\left(M\left[j\right]⊕2^{j-1}\cdot 3\cdot L\right)⊕V⊕L\right)⊕2^{i}\cdot L$ and $V=PMAC1_{K}\left(A\right)$.

In the forgery stage, A produces two forgeries $\left(A,C^{\prime }=C^{\prime }\left[1\right]||C\left[2\right]||\cdots ||C\left[d\right],T\right)$ and $(A^{\prime }=A\left[2\right]⊕17\cdot L||A\left[1\right]⊕17\cdot L||\cdots ||A\left[a\right](A\left[a\right]||10^{*}),C^{\prime },T)$, and wins as $D_{K}\left(A,C^{\prime },T\right)=M\left[2\right]⊕5\cdot L||M\left[1\right]⊕5\cdot L||\cdots ||M\left[d\right]\neq ⊥$ and $D_{K}\left(A^{\prime },C^{\prime },T\right)=M\left[2\right]⊕5\cdot L||M\left[1\right]⊕5\cdot L||\cdots ||M\left[d\right]\neq ⊥$, where $C^{\prime }\left[1\right]=E_{K}\left(E_{K}\left(M\left[2\right]⊕2\cdot 3\cdot L\right)⊕V⊕L\right)⊕2\cdot L$.

It follows that the EUF-qCMA advantage of an adversary A against COPA is

$$\begin{array}{c} Adv_{COPA}^{EUF-qCMA}\left(A\right)=Pr\left[A\;wins\right]=1. \end{array}$$

Therefore, our attack succeeds with probability 1.

## 4. COPA-ISDP: COPA with Intermediate-State Doubling Point

After careful observation, we found that 1. The IND-qCPA insecurity of COPA is due to the fact that we can construct a periodic function that calculates the intermediate state $L=E_{K}\left(0^{n}\right)$ and then use an empty associated data in the query phase to launch attacks; 2. The EUF-qCMA insecurity of COPA is due to the fact that you can control the two inputs, generating the last authentication tag to be unchanged to ensure that the last authentication tag is unchanged.

To fix post-quantum security of COPA, we require that associated data used in all stages cannot be empty and introduce an intermediate state doubling-point technique into COPA to break the intermediate state or authentication tag invariance. We refer to it as COPA with an intermediate-state doubling-point (COPA-ISDP). COPA-ISDP makes only minor changes on the basis of COPA to ensure that it inherits all the benefits of COPA. The so-called intermediate-state doubling-point (ISDP) technique uses the double-multiplication operation on a finite field to intermediate states, so that the operation breaks the quantum insecurity of direct XOR construction (ISDP induces provable algebraic nonlinearity, which is sufficient to disrupt quantum period-finding attacks while maintaining efficiency).

**Figure 2:**
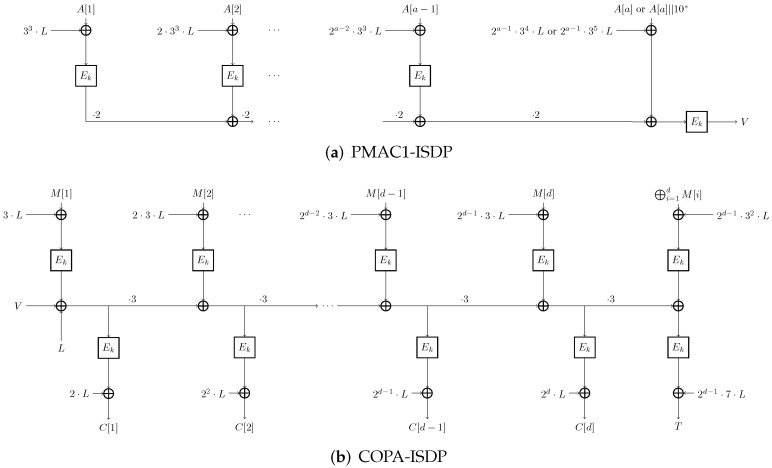
The overview of COPA-ISDP.

The overview of COPA-ISDP is shown in Figure 2. The PMAC1-ISDP, encryption, and decryption algorithms of COPA-ISDP are respectively shown in Algorithms 4, 5 and 6.

    **Algorithm 4** PMAC1-ISDP Algorithm: $PMAC1-ISDP_{K}\left(A\right)$
**Input:** Key *K* and associated data *A*
**Output:** MAC tag *V*
Partition *A* into A[1]∥⋯∥A[a], where |A[i]|=n,1≤i≤a−1,0<|A[a]|≤n
for i=1 to a−1
       $S\left[i\right]\leftarrow E_{K}\left(A\left[i\right]⊕2^{a-2}\cdot 3^{3}\cdot L\right)$
if |A[a]|=n
       $\Sigma \leftarrow 2^{a}\cdot S_{1}⊕2^{a-1}\cdot S_{2}⊕\cdots ⊕2\cdot S_{a-1}⊕A\left[a\right]$
       $V=E_{K}\left(\Sigma ⊕2^{a-1}\cdot 3^{4}\cdot L\right)$
else
       $\Sigma \leftarrow 2^{a}\cdot S_{1}⊕2^{a-1}\cdot S_{2}⊕\cdots ⊕2\cdot S_{a-1}⊕A\left[a\right]10^{*}$
       $V=E_{K}\left(\Sigma ⊕2^{a-1}\cdot 3^{5}\cdot L\right)$
**return** *V*


    **Algorithm 5** The encryption algorithm: $COPA-ISDP.E_{K}\left(A,M\right)$
**Input:** Key *K*, associated data *A*, and plaintext *M*
**Output:** Ciphertext *C* and tag *T*
Partition *M* into M[1]∥⋯∥M[d], where |M[i]|=n,1≤i≤d


$V=PMAC1-ISDP_{K}\left(A\right)$




$L=E_{K}\left(0^{n}\right)$


for i=1 to *d*
        $S\left[i\right]\leftarrow E_{K}\left(M\left[i\right]⊕2^{i-1}\cdot 3\cdot L\right)$
        $C\left[i\right]\leftarrow E_{K}\left(3^{i-1}\cdot \left(V⊕L⊕S\left[1\right]\right)⊕3^{i-2}\cdot S\left[2\right]⊕\cdots ⊕S\left[i\right]\right)⊕2^{i}\cdot L$


C←C[1]C[2]⋯C[d]




$\Sigma_{1}\leftarrow 3^{d}\cdot \left(V⊕L⊕S\left[1\right]\right)⊕3^{d-2}\cdot S\left[2\right]⊕\cdots ⊕3\cdot S\left[d\right]$




$\Sigma_{2}={⨁}_{1\leq i\leq d}M\left[i\right]$




$T=E_{K}\left(E_{K}\left(\Sigma_{2}⊕2^{d-1}\cdot 3^{2}\cdot L\right)⊕\Sigma_{1}\right)⊕2^{d-1}\cdot 7\cdot L$


**return** C||T


    **Algorithm 6** The decryption algorithm: $COPA-ISDP.D_{K}\left(A,C||T\right)$
**Input:** Key *K*, associated data *A*, ciphertext *C*, and tag *T*
**Output:** Plaintext *M* or ⊥
Partition *C* into C[1]∥⋯∥C[d], where |C[i]|=n,1≤i≤d


$V=PMAC1-ISDP_{K}\left(A\right)$




$L=E_{K}\left(0^{n}\right)$


for i=2 to *d*
       $U\left[i\right]\leftarrow E_{K}^{-1}\left(C\left[i\right]⊕2^{i}\cdot L\right)$
       $M\left[i\right]\leftarrow E_{K}^{-1}\left(U\left[i\right]⊕3\cdot U\left[i-1\right]\right)⊕2^{i-1}\cdot 3\cdot L$


$U\left[1\right]\leftarrow E_{K}^{-1}\left(C\left[1\right]⊕2\cdot L\right)$




$M\left[1\right]\leftarrow E_{K}^{-1}\left(U\left[1\right]⊕V⊕L\right)⊕3\cdot L$




M←M[1]M[2]⋯M[d]




$\Sigma_{1}=3\cdot U\left[d\right]$




$\Sigma_{2}={⨁}_{1\leq i\leq d}M\left[i\right]$




$T^{\prime }=E_{K}\left(E_{K}\left(\Sigma_{2}⊕2^{d-1}\cdot 3^{2}\cdot L\right)⊕\Sigma_{1}\right)⊕2^{d-1}\cdot 7\cdot L$


if $T^{\prime }=T$
       **return** *M*
else
       **return** ⊥


Next, we present the post-quantum security analysis of COPA-ISDP. We prove that COPA-ISDP ensures IND-qCPA security and EUF-qCMA security if the underlying block cipher is a secure qPRP or qPRF.

**Theorem** **1.** *Let A be an IND-qCPA adversary against COPA-ISDP which makes q encryption and challenge queries in total, with d being the maximum length (in blocks) of messages in each query. Let σ=q(a+2d+2). Then there exists a qPRP adversary B against the underlying block cipher $E_{K}$ with at most σ quantum queries such that*

$$\begin{array}{c} Adv_{COPA-ISDP}^{IND-qCPA}\left(A\right)\leq Adv_{E}^{qPRP}\left(B\right)+2q\sigma \sqrt{2^{-n}+\frac{2q}{2^{n/2}}}+\frac{2q}{2^{n/2}}. \end{array}$$

Proof: The security proof includes two steps. First, we replace $E_{K}$ with a random permutation $P\overset{\$}{\leftarrow }Perm\left(n\right)$, which costs

$$\begin{array}{c} Adv_{COPA-ISDP}^{IND-qCPA}\left(A\right)\leq Adv_{E}^{qPRF}\left(B\right)+Adv_{COPA-ISDP[P]}^{IND-qCPA}\left(A\right). \end{array}$$

Then, consider an adversary A playing an IND-qCPA game with COPA−ISDP[P]. In the challenge phase, A picks two classical plaintext-associated data pairs $\left(M_{0},A_{0}\right)$ and $\left(M_{1},A_{1}\right)$ with d+a being the maximum length, after which the challenger picks a random bit *b* and gives $\left(C_{b},T_{b}\right)$ to A.

We utilize the hybrid argument and define a sequence of hybrid games.

$G_{0}$: COPA-ISDP[P] with *q* superposition queries.

$G_{1}$: Firstly, we replace *V* with $V\overset{\$}{\leftarrow }{\left\{0,1\right\}}^{n}$ for each superposition query.

$G_{2i}$: We then replace C[1]||⋯||C[i] with ${\left\{0,1\right\}}^{ni}$ for each superposition query, where 1≤i≤d and $G_{20}=G_{1}$.

$G_{3}$: We replace *C* with $C\overset{\$}{\leftarrow }{\left\{0,1\right\}}^{dn}$ for each superposition query.

$G_{4}$: Finally, we replace *T* with $T\overset{\$}{\leftarrow }{\left\{0,1\right\}}^{n}$ for each superposition query.

In $G_{4}$, the distribution of $\left(C_{b},T_{b}\right)$ is independent of the distribution of the responses received by A during the query phase. Since *b* is a random bit, if $b^{\prime }$ is the bit output by A, the probability that $b=b^{\prime }$ is always 1/2. Thus, one has

$$\begin{array}{cc} Adv_{COPA-ISDP[P]}^{IND-qCPA}\left(A\right)= & \left|Pr\left[A\;wins\right]-\frac{1}{2}\right|\\ \leq & \left|Pr\left[A^{G_{0}}=1\right]-Pr\left[A^{G_{1}}=1\right]\right|\\ \; & +|Pr\left[A^{G_{1}}=1\right]-Pr\left[A^{G_{3}}=1\right]|\\ \; & +|Pr\left[A^{G_{3}}=1\right]-Pr\left[A^{G_{4}}=1\right]|\\ \leq & \left|Pr\left[A^{G_{0}}=1\right]-Pr\left[A^{G_{1}}=1\right]\right|\\ \; & +\sum_{0\leq i\leq qd}\left|Pr\left[A^{G_{2(i+1)}}=1\right]-Pr\left[A^{G_{2i}}=1\right]\right|\\ \; & +|Pr[A^{G_{3}}=1]-Pr\left[A^{G_{4}}=1\right]|. \end{array}$$

According to the One-way to Hiding (O2H) lemma [19], we have

$$\begin{array}{c} |Pr\left[A^{G_{0}}=1\right]-Pr\left[A^{G_{1}}=1\right]|\leq 2q\sqrt{2^{-n}+\frac{2q}{2^{n/2}}},\\ |Pr\left[A^{G_{2(i+1)}}=1\right]-Pr\left[A^{G_{2i}}=1\right]|\leq 2q\sqrt{2^{-n}+\frac{2q}{2^{n/2}}},\\ |Pr\left[A^{G_{3}}=1\right]-Pr\left[A^{G_{4}}=1\right]|\leq \frac{2q}{2^{n/2}}. \end{array}$$

Therefore, the IND-qCPA advantage of A against COPA-ISDP is at least

$$\begin{array}{c} Adv_{COPA-ISDP}^{IND-qCPA}\left(A\right)\leq Adv_{E}^{qPRP}\left(B\right)+2q\sigma \sqrt{2^{-n}+\frac{2q}{2^{n/2}}}+\frac{2q}{2^{n/2}}. \end{array}$$

**Theorem** **2.** *Let A be an EUF-qCMA adversary against COPA-ISDP which makes q encryption queries in total, with d being the maximum length (in blocks) of messages in each query, and outputs q+1 valid quadruples (A,C,T). Let σ=q(a+2d+2). Then, there exists a qPRF adversary B against the underlying block cipher $E_{K}$ with at most σ quantum queries, such that*

$$\begin{array}{c} Adv_{COPA-ISDP}^{EUF-qCMA}\left(A\right)\leq Adv_{E}^{qPRF}\left(B\right)+2q\sigma \sqrt{2^{-n}+\frac{2q}{2^{n/2}}}+\frac{2q}{2^{n/2}}+\frac{q}{2^{n/3}}+\frac{q+1}{2^{n}}. \end{array}$$

Proof: The security proof includes two steps. First, we replace $E_{K}$ with a random function $R\overset{\$}{\leftarrow }Func\left(n\right)$, which costs

$$\begin{array}{c} Adv_{COPA-ISDP}^{EUF-qCMA}\left(A\right)\leq Adv_{E}^{qPRF}\left(B\right)+Adv_{COPA-ISDP[R]}^{EUF-qCMA}\left(A\right). \end{array}$$

Then, consider an adversary A playing an EUF-qCMA game with COPA−ISDP[R]. Recall that in the encryption phase, A picks *q* classical plaintext/AD pairs $\left(M^{1},A^{1}\right),\cdots ,\left(M^{q},A^{q}\right)$ with d+a being the maximum length and giving $\left(C^{1},T^{1}\right),\cdots ,\left(C^{q},T^{q}\right)$ to A.

In the query phase, according to the IND-qCPA advantage and qPRP-qPRF switching lemma, the probability that A wins is upper bounded by $2q\sigma \sqrt{2^{-n}+\frac{2q}{2^{n/2}}}+\frac{2q}{2^{n/2}}+\frac{q}{2^{n/3}}$.

In the forgery phase, A picks q+1 classical valid quadruples (A,C,T). Since *R* is a random function, the probability that D(A,C,T)≠⊥ is always $\frac{1}{2^{n}}$. Thus, one has

$$\begin{array}{c} Adv_{COPA-ISDP[R]}^{EUF-qCMA}\left(A\right)=Pr\left[A\;wins\right]=\frac{q+1}{2^{n}}. \end{array}$$

Therefore, the EUF-qCMA advantage of A against COPA-ISDP is at least

$$\begin{array}{c} Adv_{COPA-ISDP}^{EUF-qCMA}\left(A\right)\leq Adv_{E}^{qPRF}\left(B\right)+2q\sigma \sqrt{2^{-n}+\frac{2q}{2^{n/2}}}+\frac{2q}{2^{n/2}}+\frac{q}{2^{n/3}}+\frac{q+1}{2^{n}}. \end{array}$$

Theorems 1 and 2 show that COPA-ISDP supports the IND-qCPA and EUF-qCMA security if the underlying block cipher is a secure qPRF.

## 5. Conclusions and Future Work

This paper focuses on the post-quantum security of COPA, and presents the first IND-qCPA security analysis of COPA. Besides that, in order to effectively avoid the problems of quantum existential forgery attack and quantum distinguishing attack, we restrict the associated data non-emptiness, introduce a novel intermediate state doubling-point technology into COPA, and present an enhanced variant, called COPA-ISDP, to support the IND-qCPA and EUF-qCMA security. However, the universal unforgeability under quantum chosen-message-attacks (UUF-qCMAs) does not considered. We leave it as an open problem to present the UUF-qCMA attack on COPA. Besides that, this paper just considers IND-qCPA security for privacy. The qIND-qCPA and other stronger quantum security notions were presented recently. It is an interesting open problem to extend the IND-qCPA security analysis to the qIND-qCPA and other stronger quantum security analyses.

## Figures and Tables

**Table 1:** Comparison between Xu et al.’s work and our work.

Aspect	Xu et al.’s Work [26]	Our Work
Security Focus	EUF-qCMA only	IND-qCPA + EUF-qCMA
Methodology	Concrete attack	Formal reduction proofs
Technical Contribution	Vulnerability demonstration	Provably secure construction
Security Guarantees	Attack-specific	Generalized bounds
Practical Impact	Highlighted problem	Solution + design paradigm

## Data Availability

The data used to support the findings of the study are available within the article.
